# Squaring the circle: a priority-setting method for evidence-based service development, reconciling research with multiple stakeholder views

**DOI:** 10.1186/s12913-015-0958-1

**Published:** 2015-08-12

**Authors:** Rebecca Hutten, Glenys D. Parry, Thomas Ricketts, Jo Cooke

**Affiliations:** School of Health and Related Research, University of Sheffield, Regent Court, 30 Regent Street, Sheffield, S1 4DA UK; Sheffield Health and Social Care NHS Foundation Trust, St George’s Community Health Centre, Winter Street, Sheffield, S3 7ND UK; NIHR Collaboration for Leadership in Applied Health Research and Care for Yorkshire and the Humber (CLAHRC YH), 11 Broomfield Road, Sheffield, S10 2SE UK

**Keywords:** Consensus, Depression, Priority-setting, Self-care, Program development, Quality improvement, Mental health services, Decision-making, Recovery-orientation, Stakeholder engagement

## Abstract

**Background:**

This study demonstrates a technique to aid the implementation of research findings through an example of improving services and self-management in longer-term depression. In common with other long-term conditions, policy in this field requires innovation to be undertaken in the context of a whole system of care, be cost-effective, evidence-based and to comply with national clinical guidelines. At the same time, successful service development must be acceptable to clinicians and service users and choices must be made within limited resources. This paper describes a novel way of resolving these competing requirements by reconciling different sources and types of evidence and systematically engaging multiple stakeholder views.

**Methods:**

The study combined results from mathematical modelling of the care pathway, research evidence on effective interventions and findings from qualitative research with service users in a series of workshops to define, refine and select candidate service improvements. A final consensus-generating workshop used structured discussion and anonymised electronic voting. This was followed by an email survey to all stakeholders, to achieve a pre-defined criterion of consensus for six suggestions for implementation.

**Results:**

An initial list of over 20 ideas was grouped into four main areas. At the final workshop, each idea was presented in person, visually and in writing to 40 people, who assigned themselves to one or more of five stakeholder groups: i) service users and carers, ii) clinicians, iii) managers, iv) commissioners and v) researchers. Many belonged to more than one group. After two rounds of voting, consensus was reached on seven ideas and one runner up. The survey then confirmed the top six ideas to be tested in practice.

**Conclusions:**

The method recruited and retained people with diverse experience and views within a health community and took account of a full range of evidence. It enabled a diverse group of stakeholders to travel together in a direction that converged with the messages coming out of the research and successfully yielded priorities for service improvement that met competing requirements.

## Background

This paper describes a new technique for reconciling the views of multiple stakeholders with research evidence when identifying testable service improvements. The method is designed to be applicable to local service improvements and their evaluation in long-term conditions, through the exemplar of psychological therapy services for people with Longer Term Depression (LTD).

It is an example of the methods developed by the UK’s National Institute for Health Research (NIHR), Collaboration for Leadership in Applied Health Research and Care (CLAHRC) whose aim is to reduce the research-practice gap and enhance applied research that is relevant to practice and service users [1, 2].

Policy-makers experience an inherent tension when improving services and setting research priorities for testing service improvements. Policy must be ‘evidence-based’, so that improvements are grounded in research evidence and adhere to national clinical guidelines. At the same time, within the UK, there is a policy imperative to develop services that are ‘clinically led’ particularly around commissioning [3]. In addition, the patient voice and experience in shaping services and research priorities is also required [4–7]. The tension between these differing requirements is particularly acute in agreeing research priorities for whole-system service improvements of complex and long-term conditions where the implementation of national guidelines is challenging [8, 9]. For example, current UK mental health policy emphasises personalisation, partnership and co-production [10, 11] and states the importance of taking account of mental, physical, social and occupational outcomes for service users using the term ‘recovery’ to encompass broad aspects of quality of life [7]. There is increasing recognition of the challenges faced by large numbers of people whose health problems cross the long-term physical conditions-mental health divide [12], but limited evidence that services have yet developed organisational responses that meet these challenges [13, 14].

A number of methods have been used to allow the views of multiple stakeholders to inform research priorities. For example, the ‘James Lind Alliance’ (JLA) priority setting partnerships – formal partnerships of patients, carers and healthcare professionals – generate, define, refine and prioritize unanswered research questions [15, 16]. Similar methods have been used in a wide range of health topics: diabetes care [17], cancer genomics [18], maternity services [19], natural resource planning and management [20], COPD and asthma [21] and pancreatic cancer [22]. Most of these involve representative organizations being tasked with consulting their memberships or inviting participation in a survey, before taking part in formal meetings or focus groups, presentations and voting activities.

The extent to which these representative organizations include or represent service users and carers varies. Most approaches require specialist input for the preparatory briefing of participants, to distinguish unanswered research questions from those already addressed, and for the development of early ideas into researchable propositions. Some adopt economic decision modelling techniques to evaluate and present evidence (for example [23]) whilst others rely wholly on in-person methods, wherein professional and lay (especially patient and carer) ‘experience’ is voiced, debated, summarised and compared. Formal consensus generating methods such as Delphi surveys and nominal group techniques have also been used or advocated in a number of studies and reviews [22, 24, 25]. Relatively few studies have taken these techniques into the realm of mental health care; the James Lind Alliance used its systematic methods to derive research priorities in schizophrenia [26] and are currently undertaking a similar exercise in relation to depression; Gold and colleagues [27] used a combination of ‘systematic evidence reviews’ and a half day stakeholder workshop to define and assess gaps in the evidence for depression screening in adults in primary care; and Claassen [28] defined a hybrid multi-stage decision model for selecting research priorities in the area of suicide prevention.

Although these valuable initiatives have been used to generate research priorities, they have not been used to address the practical difficulties faced by services needing to achieve cost-effective and evidence-based improvements, which also address the priorities of a comprehensive range of stakeholders (including funders, providers and users). Furthermore, they do not always amount to a systematic approach to achieving a participatory process between these stakeholders [29]. Finally, they are not necessarily grounded in a formal cost-and-outcome model of a whole service system for a given condition.

### The IQuESTS programme and the role of consensus within it

The IQuESTS research programme (Improving Quality and Effectiveness of Services, Treatments and Self-management in longer-term depression http://clahrc-sy.nihr.ac.uk/theme-iquests-introduction.html) was part of a Government initiative in England designed to reduce the research-practice gap and enhance applied research that is relevant to practice and to service users. It aimed to generate actionable, innovative and evidence-based interventions to improve existing psychological treatments and services for people experiencing longer-term depression, from a position which endorsed the principles of self-management. The IQuESTS research team included service users, practitioners and academics from a range of disciplines: social science, health services research, clinical psychology, mental health nursing, health economics and mathematical modelling. The project had three stages.

The first stage involved two research projects which generated different types of relevant knowledge for service improvement: cost and effectiveness of service pathways and service-user experience of services and self-management. The first study in this stage built a mathematical model of the NHS care pathway for longer-term depression using a ‘whole disease’ framework [30]. The model synthesised research evidence with local data on process and outcomes and provided the ability to assess the potential costs and benefits of service improvements [31]. The second study in this first stage focussed on ‘learning from the patient’ using qualitative research methods to identify and explore self-management strategies used by people with experience of longer-term depression, defined as two or more episodes of major depression. It explored what services could learn from individuals’ own strategies and choices and how services could support effective methods of self-management.

It is the second stage of IQuESTS which is presented in the present paper. Results from the first stage were presented to two workshops where practitioners, managers, service users and academics reviewed the evidence and generated a ‘long list’ of candidate ideas for service improvement. Formal consensus generating methods were then used to prioritise these on the basis of pre-specified criteria, to yield a realistic number of innovations to be taken forward into a third stage, where they would be implemented in a clinical service in order to be evaluated for feasibility and acceptability.

**Fig. 1:**
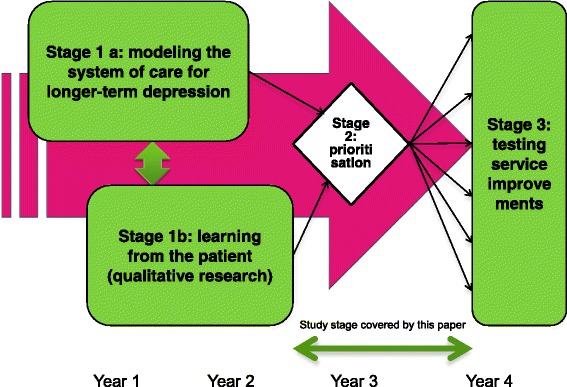
IQuESTS research and implementation process

This paper therefore describes the middle stage between stages 1 and 3, which involved a) generating candidates for improving therapies and services and b) the formal prioritisation process (see Fig. 1).

### Study aims

To demonstrate a method of generating and agreeing on service improvement priorities for people with longer-term depression which

- systematically involves a wide set of stakeholders
- locates service changes within a ‘whole-system’ model of care
- is grounded in research evidence
- addresses some of the inequalities that undermine co-production, through the use of formal consensus-generation methods, and
- generates service changes that are themselves researchable.

## Methods

In order to generate and capture ideas for service improvement, two day-long workshops were held in Stage 1 (one for each study), as preparation for Stage 2. Participation in the workshops was by invitation which resulted in 37 and 34 participants respectively. Invitees were identified by the study team using their knowledge of local and national networks of service users and carers and by approaching NHS clinicians working in primary, secondary and specialist psychological therapy services in mental health organisations within the region. Primary care doctors with a special interest in mental health, doctors and others involved with commissioning managed care and NHS managers with a service planning role were also invited. In addition, voluntary sector and NHS service providers were included as were a range of health service researchers based at local Universities.

Participants in the first (‘mathematical modelling’) workshop identified four suggestions for service improvement that could be appraised using the model. These were: self-referral back to therapist after discharge, case management (linking physical and mental health services), widening access to non-therapy services (for those in specialist care), and use of a common assessment and monitoring tool (across mental health services) to avoid unnecessary multiple assessments.

At the second workshop (‘learning from the patient’), participants identified a very long list of themes which were relevant for the improvement of services. These were grouped by the research team after the workshop under broad headings, which could be linked with research evidence and eventually re-organised and turned into 18 potential service improvement ideas. These included a range of education and guided self-help, support and treatment ideas and suggestions for complementary activities. The combined list of ideas from both workshops, along with a further five ideas based on the project team’s collective knowledge and experience of applied mental health research was then put forward for consideration by the project Steering Group.

In total 27 service improvement ideas were available for review at this point, and a pro forma was completed for each idea, describing what it was, the evidence to suggest it might be helpful, any comments on the feasibility of implementation and notes on how its impact might be assessed. The Steering Group’s task was to identify overlaps, ‘non-starters’ and gaps in the list. Several ideas were combined and a revised total of 20 ideas were put forward for development and presentation at a third workshop, the next step in the prioritisation process.

Table 1 gives the suggestions for service improvement generated and the workshop or meeting source each came from.

**Table 1:** Actionable service improvement ideas generated from projects in first stage of the IQuESTS research programme

No.	Title and description of actionable service improvement	Source of idea
*Education and guided self-help ideas*		
1	Widening access to non-therapy services – to help service users build and strengthen their support networks by engaging (or re-engaging) with activities and/or services they valued in the community.	Stage 1a
2	Educating staff and families about depression – to provide psycho-educational materials and training opportunities to friends, family, and carers of people with longer-term depression as well as professionals.	Stage 1b
3	Guided self-help with the use of tools and resources – helping patients to navigate the wealth of existing written and on-line information and self-help resources available.	Stage 1b
4	Sign-posting & improving access to a menu of options alongside therapy including, for example:	Stage 1b
	• Pets for Companions	
	• Complementary Therapies	
	• Voluntary work or job search activity	
	• Physical exercise	
*Support and treatment ideas (within psychotherapy services)*		
5	Peer buddy programme – developing one-to-one peer mentoring to help with initial engagement and to run alongside therapy.	Stage 1b
6	Improved brokerage/ensuring informed choice pre-therapy start – focussing on quality of information and communication about available choices prior to therapy.	Stage 1b
7	Peer Support Group post treatment – professionally facilitated and peer managed support groups to reinforce and maintain the gains made in therapy.	Stage 1b
8	Motivational Interviewing and Goal setting – using these as a specific technique/intervention within therapy.	Stage 1b
9	Mindfulness based relapse prevention – professionally led, group-based training in mindfulness techniques at the end of therapy.	Steering Group/Core Team meeting
10	Wellness Recovery Action Plan (WRAP) & relapse prevention – using the WRAP tool as a specific intervention in relapse prevention planning.	Stage 1b
*Care pathway related ideas*		
11	Self-referral back to therapist after discharge – enabling service users to access the same or a different therapist without professional re-referral after completion of therapy.	Stage 1a
12	Integrating care co-ordination and psychological services – providing case management for people using social care and health services.	Stage 1a
13	Developing a common assessment and monitoring tool for use across the care pathway – to reduce the burden on service users of providing the same information repeatedly across mental health services.	Stage 1a
14	Physical health reviews/physical health link workers – conducting physical health assessments alongside mental health reviews for psychotherapy patients, to ensure physical health needs are not neglected.	Steering Group/Core Team meeting
15	Improving access for Black and Minority Ethnic (BME) people into services – developing and increasing outreach and engagement activities with BME communities.	Steering Group/Core Team meeting
16	Better management and prevention of drop-out – using evidence-based techniques (intention implementation planning) to minimize non-attendance during therapy.	Steering Group/Core Team meeting
*Complementary Activities*		
17	Green activities – promoting volunteering in conservation and allotment gardening.	Stage 1b
18	Help to get started and continue doing things – providing occupational therapy assistance for people to help with routine activities of daily living.	Stage 1b
19	Incorporating balance of activities and routines - encouraging a focus on balance of activities in developing new routines.	Stage 1b
20	Work for well-being – investigating and taking up opportunities for voluntary and paid work.	Stage 1b

The aim of the decision-making and consensus generating element of IQuESTS was to reduce the long list of 20 ideas to a final six ‘actionable’ service improvements which could be introduced and evaluated in practice (in Stage 3). Consensus was reached through a multi-stakeholder workshop using a modified nominal group technique, followed by a Delphi email survey. Combining these two methods was intended to maximise participation and inclusion.

In total, views were sought from 104 participants who were asked to become involved in one or more of the consensus activities, and to ascribe themselves to one (or more) of five stakeholder groups: (i) clinician/practitioner, (ii) service provider/clinical manager, (iii) service user/carer/ advocate, (iv) service commissioner or planner, (v) health service researcher (see Tables 2 and 3). These individuals were recruited from the list of Stage 1 participants, with additional participants identified by project steering group members to ensure coverage across the stakeholder groups, in light of job changes and other individual changes of circumstance. Two thirds were women.

**Table 2:** Stakeholders represented in the consensus workshop

Stakeholder group	Number (identifying with this category as ‘main group’)	Of which, number belonging to 2 or more groups
Service users/carers/advocates	10	2
Clinicians/practitioners	14	5
Clinical managers	3	1
Commissioners	2	0
Health service researchers	11	3
Total	40	11

**Table 3:** Stakeholders represented in the email survey

‘Main’ stakeholder group	Email survey (number identifying with this category as ‘main group’)	Of which, number belonging to 2 or more groups
Service users/carers/advocates	5	1
Clinicians/practitioners	19	12
Clinical managers	3	1
Commissioners	3	1
Health service researchers	9	4
Total	39	19

The consensus workshop was a whole day event. Participants were prepared for the workshop through a detailed briefing pack sent a week in advance which described the process and the purpose of the workshop, and included descriptions of each of the 20 candidate ideas. Participants were invited (but not required) to canvass views from colleagues in advance using this written material. The ideas were further described during the workshop itself through a series of short presentations followed by a question and answer session. Each idea had a ‘champion’ from the study team to present the evidence for the idea, and to answer questions on it. Ideas were presented in four batches of linked approaches targeting particular aspects of the care pathway for longer-term depression. These were grouped as follows: education and guided self-help ideas; support and treatment ideas; care pathway ideas, and complementary activities.

Workshop participants were asked to consider each idea based on criteria given in Table 4, which were selected to represent a balanced set of requirements for an actionable implementation. In making a choice, participants were asked to favour improvement ideas which, in their opinion, were most likely to meet the criteria. It was stressed that each person’s view had equal weight, in reaching a collective decision and that, for example, a proposal that had a strong evidence base but was considered unacceptable to service users would not be favoured over one that met both criteria.

**Table 4:** Criteria used to assess candidate ideas in consensus workshop

1. Is it a useful idea?	
- How strong is the evidence?	
- Will it help and be acceptable?	
2. Is it feasible?	
- How practical and realistic is it to implement?	
3. Can we assess the impact?	
- Will it be measurable through the methods proposed?	
- Will additional measures be needed?

**Table 5:** Prioritisation scores across two stages of consensus process

Rank/Idea No.	Idea Name	Consensus Workshop		Email Survey	
		N	%	N	%
1.	Help to get started & continue doing things (behavioural activation)	33	82	31	80
2.	Mindfulness based relapse prevention	34	86	28	72
3.	Work for well-being	35	87	27	69
4.	Better management & prevention of drop-out	33	82	25	64
5.	Widening access to non-therapy services	35	88	25	64
6.	Self-referral back to therapist after discharge	33	83	22	56
7.	Green activities	31	78	21	54
8.	Physical health reviews	33	82	21	54

Workshop participants were then asked to vote using anonymised electronic voting technology to identify their own individual priorities. They were not required to formally ‘represent’ their employing organisations but were asked to respond as themselves, drawing on their own experience and knowledge base (the ‘expert by experience’ perspective). Voting was on a scale from 1 to 9, with 1 being the lowest priority, 5 moderate priority and 9 high priority. Participants were asked to use the full range of available scores to represent their opinion. Each person had one vote for each idea. Consensus was pre-defined as being reached when 80 % of votes cast scored 7 or above for that idea. Feedback on the first round of voting was provided immediately to the workshop participants. Active debate then ensued to discuss the pros and cons of each idea in order to work towards consensus. Contributions from all participants were welcomed. Those who were less confident to speak out during the debate were invited to write feedback down. Other facilitation techniques supported inclusion and space to contribute, for example, careful attention was paid to the lay-out of the room, the scheduling of the day (clear timing and frequent breaks) and to recognise different communication styles and preferences (use of display materials, practice voting questions and setting ‘ground rules’ for discussion). All stakeholder groups asked questions as the day progressed. The service user groups were particularly vocal.

After the debate a second round of electronic voting was undertaken. The overall participation rate in both rounds of voting was 95 %, and there were minor variations in the total number of votes cast for each idea. This produced consensus on eight ideas.

These were then used in the email survey to the full sample (*n* = 104) of stakeholders (37 men, 67 women). The questionnaire was sent out along with a brief description of the eight ideas to be prioritised. Participants were asked to rate these ideas using the same evaluation criteria and scoring system as in the workshop. Only one questionnaire round was undertaken due to constraints of time within the research programme. Participants were given three weeks in which to return the survey and one email reminder was sent. Scoring results from the Consensus workshop were included in the questionnaire so that respondents could see how priorities had been scored previously.

### Ethics

All component studies of the IQuESTS programme were ethically reviewed and approved by the NHS Research Ethics Service (NRES) for Yorkshire and the Humber South Yorkshire committee. Stages 2 and 3 were combined in application reference 11/YH/0392. Informed consent was sought and repeatedly renewed throughout the research process and all workshop and email survey participation was voluntary.

## Results

### Moving from 20 ideas to eight: the Consensus Workshop

Of the 104 people identified and invited to attend the workshop, 50 agreed to take part and 40 were able to attend on the allotted day. Of these 40 individuals, 20 had not previously taken part in a Stage 1 workshop and were new to the study. The majority of participants identified with a single stakeholder group (63 %) with another 20 % identifying with two.

#### First round of electronic voting

**Fig. 2:**
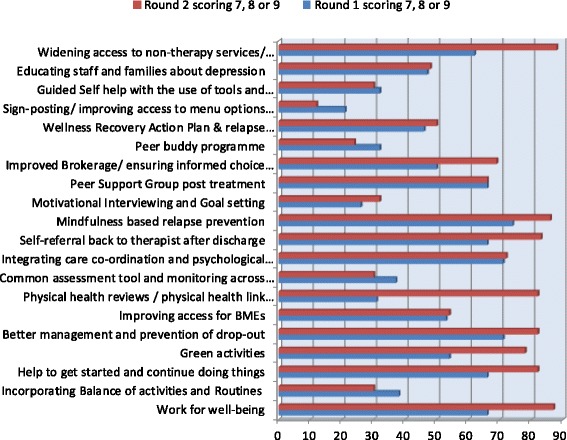
Voting results from consensus workshop

Following the first round of voting no single idea achieved consensus. Although encouraged to use the full nine-point scale, some workshop participants appeared reluctant to conclusively rule in or rule out ideas by giving an unequivocally high or low score. Instead, there was a good spread of support across the 20 ideas, with a mixture of people both strongly supporting and not supporting a few ideas, but generally indicating moderate support using the mid-range of available scores for most ideas (see Fig. 2 for details).

This finding proved useful in galvanizing participants to ask more questions and to challenge each other during the plenary debate which followed Round 1 of voting. Through this process, idea champions and workshop participants who supported or had strong views on particular ideas were motivated to articulate more clearly the potential strengths and weaknesses of their idea. A fruitful and informative debate followed, in which supporters and detractors of ideas spoke with more passion and eloquence than they had during the first discussion and more people engaged in the discussion.

#### Second round of electronic voting

Participants thus entered the second round of voting. This time seven out of the 20 ideas achieved a consensus rating of 80 % or higher. Two further ideas received ratings of 78 % (‘Green activities’) and 72 % (‘Integrating care co-ordination and psychological services/case management’) respectively. As there were three ideas clustered around the mid 60 % mark, it was decided to include only the higher rated of the two runner up ideas in the list to be voted on via email survey – making a total of eight shortlisted ideas.

#### Email questionnaire

Thirty nine of the 104 participants previously invited to workshops responded to the email survey (response rate 38 %). Of these, four had not previously participated in a Stage 1 workshop, and 12 had not attended the consensus workshop. Participants were asked again to self-ascribe themselves to multiple and then a ‘main’ stakeholder group, as shown in Table 3. Just over half (51 %) identified with a single stakeholder group and 44 % identified with two.

The email survey completed the process of prioritisation (in effect a third round of voting), by confirming the ranking of each of the six top rated ideas, and the two lowest rated which would not go forward for implementation. The ordered list of priorities following both the consensus workshop and postal survey was thus (Table 5).

### Detailed analysis of voting responses

There was a significant positive correlation between Round 1 & Round 2 of the electronic voting, in terms of scores (*Pearsons r: .82 p < 0.001)* and rankings (*Spearmans rho: .74, p < 0.001)*.

The top six (and eventually chosen) ideas in Round 2 were ranked in the top eight at Round 1. Of the top eight ideas in Round 1, one received the same score in Round 2, while another increased by just one point. The remaining six (that were top ranked in Round 2) increased their scores by between 11 and 26 points. Three ‘not chosen’ ideas increased their votes by similar amounts but as they had a lower starting point, did not move into the top six. The most dramatic increase in this group was for ‘Physical health reviews’ rising from 31 % to 82 % consensus; the second positive change was for ‘Widening access to non-therapy services’ (rising from 62 % to 88 %) and the third for ‘Green activities’ (54 % to 78 %).

The mean (sd) score in Round 1 for those ideas eventually chosen (after Round 2) was 67.5 (4.28) compared with 43.1 (14.8) for those not chosen (significant at p < 0.001). This suggests a relatively high level of agreement early on regarding the value of the top six ideas eventually chosen, and as might be expected, less agreement overall about the remaining ideas.

The mean (sd) scores in Round 2 were 84.7 (2.66) and 48.4 (22.4) for those chosen vs not chosen respectively. This difference was also significant (p < 0.001). This would indicate that the overall level of agreement increased in this round, and increased substantially for the top six eventually chosen ideas. This suggests the discussion between the first and second rounds of voting at the workshop was effective in increasing overall consensus across all stakeholder groups in favour of the most highly rated ideas. The impression given at the time that people were being cautious in scoring in Round 1 is confirmed by the fact that the spread (range) of scores in Round 1 was narrower than in Round 2 (21-74 compared with 12 – 88). Thus people did respond to seeing the inconclusive results of the first round of voting by scoring their preferred ideas more determinedly and boldly in Round 2.

The email survey confirmed the ranking of the top six ideas from the shortlist of eight service improvement ideas put forward. Participants in the survey (as in the individual workshops) overlapped substantially but not wholly with the Consensus workshop participants. Thirteen who had taken part in the workshop did not respond to the survey, and 12 who had not been able to attend the workshop did complete the survey.

The surprising result in this round was the drop in support for physical health reviews, which dropped from a score of 82 % to a below-consensus score of 54 %. This was one of the ideas on which there was particularly impassioned debate after Round 1 voting in the Consensus workshop. It’s possible that the idea was therefore harder to evaluate in written form, and needed the dynamic of face to face interaction to bring it to life. It thus lost out from the change of voting vehicle at this stage.

## Discussion

We set out to demonstrate a method of generating and agreeing research based service development priorities for people with longer-term depression which systematically involves a wide set of stakeholders; locates service changes within a ‘whole-system’ model of care; is grounded in research evidence; and addresses some of the inequalities that undermine co-production, through the use of formal consensus-generation methods. In contrast to many other reported health research priority setting studies [19, 22], built into this process was an opportunity for the early evaluation of the applied interventions selected.

With regard to the composition of the stakeholder groups, one third of the workshop participants and a half of the survey respondents belonged to more than one group. It is possible that this reduced the diversity of the sample, but is also arguably more representative than choosing people with only one identification, in that many service providers are also service users, researchers are often clinicians, and so on. The unequal size of the groups reflected the way we defined them (based on one ‘main’ group); for example, the group ‘Commissioners and planners’ and ‘Clinical/service managers’ were much smaller than the others, and could have been combined. The largest groups represented were clinicians/practitioners and health services’ researchers. Service users/carers & advocates formed the mid-size of stakeholder group in each round of decision-making. The process of recruiting and retaining stakeholders with diverse experience and expertise was challenging. Given the prevalence of mental health problems in the population it was unsurprising but important to allow multiple roles to be identified within the process of self-categorisation.

There was overlap in participants across all three IQuESTS workshops, which enabled some shared learning to take place. Participants became better known to each other and to members of the research team through the time spent together in workshops and intervening activities. It was helpful for all groups, but particularly service users and carers, to be able ‘buddy up’ with others to ask questions of those who were more familiar with the project, and space was made for private and small group conversations to happen before each plenary debate. The use of ‘ground rules,’ ‘countdown’ cards to support time-keeping and a relaxed and welcoming style of chairing, enabled the plenary discussions to remain focussed and good humoured. This was confirmed in informal feedback after the event from participants in all groups, but especially service users – who specifically commented on having enjoyed the day, the opportunity to participate and the quality and inclusiveness of discussion. It would appear that the process did engage the range of stakeholders, and that both the achievement of pre-determined consensus levels and positive feedback from participants would indicate that all groups were enabled to participate in a meaningful way.

A novel aspect of the IQuESTS programme was the inclusion of the ‘whole-system’ modelling approach alongside the intensive qualitative research with longer-term depressed people. Presenting the results of the modelling work in lay language during the first and third workshops was challenging. Feedback from service users and carers who attended the first workshop told us we hadn’t got it quite right; we needed to make our presentations shorter and less technical and allow more time to ask questions. We listened to this, and adopted a ‘balloon debate’ style of presentation in the third ‘Consensus workshop’ – with each idea champion summarizing key points in less than 5 min. We also made use of posters, display materials and information packs, which could be consulted throughout the day, especially during breaks.

The use of whole systems modelling data were invaluable in addressing questions of potential cost-effectiveness of service improvements, enabling a better understanding of the impacts of service changes on each other. Being able to work from a shared visualisation of the whole system’s patient pathways was in itself a revelation to the different stakeholder groups. The addition of cost/outcome simulations enabled realistic understanding of the need to make choices, and jointly-focussed attention on the need to balance investment of a finite resource with evidence of effectiveness.

Overall, the results of the IQuESTS prioritisation process are closely linked both to policy priorities and evidence. The top chosen service improvement ideas fit well with the recovery agenda in mental health, in requiring an emphasis on personalisation, meaningful activity and relationships and a more positive approach to living with a chronic condition [32]. Most of the ideas were firmly grounded in the recovery and self-management literature [33, 34] as well as in the empirical research and system modelling work carried out in the first stage of the IQuESTS programme.

There was 100 % support from the three smaller stakeholder groups for ‘Widening access to non-therapy services,’ ‘Mindfulness based relapse prevention’ and ‘Help to get started and continue doing things.’ The only idea which received less than two thirds support amongst service user/carers and advocates, was ‘Prevention and management of drop-out.’ The top rated idea following the email survey (‘Help to get started and continue doing things’) was presented and developed specifically by service-user researchers in the project team. We are thus confident that no stakeholder group was excluded and all groups’ views counted in the final decision-making.

The main benefit of the prioritisation process adopted was to enable a diverse set of stakeholders to travel together in a direction that converged with the messages coming out of the research. Gold et al. [27] have highlighted the challenges of engaging clinician, patient and public stakeholders with the products of systematic evidence reviews [27]. In presenting and drawing upon feedback from stakeholders at each stage, we were able to reconcile the competing demands of being evidence-based, clinician and end-user led. In addition, the use of formal consensus methods, which enabled individuals to vote anonymously, and to review their decisions over several rounds proved supportive of all stakeholders. The process also greatly helped us to win support for implementation during Stage 3 of IQuESTS. All six ideas were developed in continuing consultation with stakeholders, and manualised prior to testing in a Specialist Psychotherapy Service for people with longer-term depression. This final stage of implementation would not have been possible if we had not harnessed the potentially competing requirements of evidence, consistency with clinical guidelines and the strongly held personal and professional views of those with lived experience in and around depression-focussed services.

### Limitations of the study

The process for evidence based service development was relatively time-intensive and related to only one aspect of mental health service provision, care for people experiencing longer-term depression. Whilst all aspects of the process (collaborative identification of quality improvement ideas; workshop-based review & refinement; decision-making workshop; email survey) appeared to add value to the engagement and outcomes, more time efficient approaches that achieve similar levels and quality of engagement may be an important refinement. The variable levels of engagement with different aspects of the process by different stakeholder groups would indicate both the benefits and challenges of maintaining engagement over time, and the need for multiple approaches. Half of the participants in the workshop had not previously participated in a Stage 1 workshop; whilst only 10 % of the email survey respondents were taking part for the first time. This suggests it is difficult to engage people in a meaningful way without face-to-face interaction, and confirms our view that the email survey was a confirmatory rather than discrete part of the decision-making process. Introducing new participants enabled the membership of ‘stakeholder’ groups to be refreshed, and the overall number of views considered to be increased, however there was some inevitable loss in the quality of engagement, as respondents to the email survey could only provide brief written comments, in addition to a score, for each idea. Having a common core of participants across all components and stages helped the collective learning process, but limited the numerical size of the sample. In contrast with approaches used by the James Lind Alliance (15,16), participants were not required to formally represent their employing organisations or stakeholder group, but to respond as experts by experience. It is unclear how this approach to representation affected respondent choices.

A possible source of bias is that some individuals participated at multiple phases while others only participated in one stage of the process. Thus, those individuals who attended all workshops and completed the email survey had more opportunities to vote and were arguably more influential than those who were less involved. However, there is no evidence that level of involvement systematically varied with stakeholder group, and the nature of the decision-making at each stage (requiring a consensus across 80 %) mitigates against untoward influence of a subgroup.

Resource and data limitations meant that only three of the 20 service improvement ideas presented at the Consensus workshop and email survey could be specifically appraised using the whole system modelling work [31]. The ability to appraise ideas in this way depended on the availability of prior research and service evaluation data to make the modelling meaningful. This limitation thus had the potential to bias some participants in favour of those ideas for which relevant data were available. All three ideas that were subject to modelling work (Better management & prevention of drop-out; Widening access to non-therapy services; Self-referral back to therapist after discharge) were amongst the final six service improvements chosen for clinical piloting.

## Conclusions

Consensus processes are under-exploited in service development and complex organisational change. We have presented evidence that they can help answer questions about priorities, particularly in areas where resources are limited and complex service improvements are required. We suggest that they are particularly valuable where practitioners and service users would otherwise be unconvinced by research evidence on cost-effectiveness and feel disenfranchised by service change based solely on this. The present study demonstrates a useful method for translating research findings into a complex service delivery system in a way which is likely to be both cost-effective and have wide-ranging support from service users, providers and funders.

### Availability of supporting data

The raw data for Fig. 2 and copies of all supporting written documents, presentations and questionnaires used in the research are available as PDFs on request, from the corresponding author.

## Abbreviations

BME: Black and Minority Ethnic group
CLAHRC: Collaboration for Leadership in Applied Health Research and Care
COPD: Chronic obstructive pulmonary disease
IQuESTS: Improving the quality and effectiveness of services, therapies and support for self-management
JLA: James Lind Alliance
LTD: Longer term depression
NHS: National Health Service
NIHR: National Institute for Health Research
NRES: National Research Ethics Service
